# Family Sibling Effect and Executives’ Corporate Social Behavior

**DOI:** 10.3389/fpsyg.2021.667529

**Published:** 2021-06-15

**Authors:** Minna Zheng, Guangqian Ren, Lingling Zhuang

**Affiliations:** ^1^Business School, Nankai University, China Academy of Corporate governance, Tianjin, China; ^2^Business School, Zhengzhou University, Zhengzhou, China

**Keywords:** family sibling interaction, executives’ sibling number, corporate social responsibility behavior, birth order, female sibling

## Abstract

Corporate social responsibility is an important business strategy for enterprises. Scholars have conducted much beneficial research on the relationship of executives’ recognitive traits and firms’ CSR behavior, but rarely focus on the impact of executives’ early recognitive traits derived from family sibling interaction. This paper takes Chinese A-shared private listed companies from 2014 to 2017 as the research samples to investigate the effect of the number of executives’ siblings on the early family sibling and corporate social responsibility behavior. We further study the moderating effect of birth order and gender composition in siblings on this relationship. The results show that there is an inversed U-shaped relationship between the number of executives’ siblings and corporate social responsibility behavior. Further research shows that the relationship between the number of executives’ siblings and CSR behavior is strengthened when an executive is first-born or has female sibling(s).

## Introduction

An emerging study has begun to pay more and more attention to the influence of executives’ recognitive traits on enterprises’ decision-making. The important influence of early family interaction on individual cognitive formation, personality, and behavioral preference has widely concerned scholars in the field of social psychology. As a kind of kinship relationship with the longest duration in human beings (Whiteman et al., 2011), sibling relationship is considered to be an important factor in predicting adult psychological behavior (Taubman-Ben-Ari, 2018). Therefore, it is of great significance to focus on the influence of original family life interaction on executives’ behavior styles and decision-making preference (Slomkowski et al., 2001).

Some studies have highlighted the impact of the sibling on multiple aspects of an individual’s behavior, including educational achievement (Booth and Kee, 2009; Weng et al., 2019), smoking behavior (Juon et al., 1995; Slomkowski et al., 2005), driving style (Taubman-Ben-Ari, 2018), etc. A recent study by Campbell et al. (2019) proposed that executives’ birth order would affect the strategic risk behavior of the enterprises in which the executives worked for, especially emphasizing the important influence of the early family sibling effect on executives’ business behavior.

However, there is still less research on the influence of executives’ siblings on their prosocial behaviors and corporate social responsibility performance. This paper aims to fill this research gap and study the impact of executives’ sibling number in the early family on the executives’ CSR behavior through influencing the early cognitive formation process. Due to the fact that corporate behaviors are the reflection of top executives’ unique personalities and backgrounds (Hambirck and Mason, 1984), executives’ prosocial preferences directly influence their strategic choices and firms’ CSR behaviors (Hambrick, 2007).

This paper takes Chinese non-financial private listed companies from 2014 to 2017 as the research samples and empirically tests the relationship between the number of executives’ siblings and the firms’ CSR behavior. We also investigate the moderating effect of birth order and sibling gender composition. The results show that there is an inversed U-shaped relationship between executives’ sibling number and firms’ CSR behavior. Further research found that when an executive is the oldest among siblings or has female sibling(s), the relationship between the number of executives’ siblings and the CSR behavior is further strengthened.

This paper mainly contributes to three aspects: First, it enriches the relevant research in the field of social responsibility, extends the research on personal family factors in the field of social psychology to the research on corporate social responsibility, and explores a new driving factor of corporate social responsibility behavior from the perspective of executives’ early family domain. This paper further verifies the influence of the sibling effect in the family environment of the executives’ early family on CSR behavior in adulthood. Secondly, the upper echelon theory is further expanded. The existing research focuses on executives’ demographic characteristics, cognitive characteristics, work experience, etc. On the basis of tracing executives’ childhood family effects on their behavior and decision preference, this paper contributes to a more comprehensive understanding of how executives’ personal cognitive differences derive from their early family life. Third, this study promotes interdisciplinary research. We extend the research on family sibling effect from the field of social psychology to the business practice, which promotes cross-discipline research.

The research arrangement of this paper is as follows: The second part is the theoretical foundation and research hypothesis. The next part proposes the data and methodology. The fourth part reports the empirical analysis results, and the last part is the research conclusion and discussion of this paper.

## Theory Foundation and Hypothesis Development

### Executives’ Sibling Numbers and Corporate Social Responsibility Behaviors

The number of siblings directly affects the investment of parents and the allocation of family resources, as well as whether children are treated equally in the family (Blake, 1981). The distribution of family resources and parents’ attitudes toward children may greatly influence the quality of sibling relationships. Harmonious and conflictual interactions coexist in sibling relationships (Deater-Deckard et al., 2002), and what kind of sibling interactions (harmony or conflict) are dominant is closely related to their prosocial behavioral tendencies (Harper et al., 2016). Early family sibling experiences determine individuals’ behavioral decisions during the childhood and even the whole life-span (Suitor and Pillemer, 2007).

Under the conditions of limited family income and resources, while there is a smaller number of children, parents have enough time and energy to care for their children, and the household resources can also meet their self-needs. In this case, sibling rivalries involving family resources are relatively weaker (Booth and Kee, 2009). Therefore, prosocial behaviors such as sharing, helping, affection, and giving are more likely to be exhibited among siblings (Dunn and Munn, 1986; Recchia and Howe, 2009), so it is easily to form a warm and harmonious sibling relationship. The early family experience of harmonious interaction among siblings in childhood makes them more likely to consider the feelings of others with empathy and affection, and promotes self-regulation and prosocial behavior of individuals (Padilla-Walker et al., 2010).

However, the amount of family resources available to each child will gradually decrease with the increase of the sibling number, and the competition and conflict for parents’ attention and family resources may be intensified (Booth and Kee, 2009; Weng et al., 2019). When there is a larger number of children, siblings have to compete for the parents’ attention, time, and household resources (Whiteman et al., 2011). Under this circumstance, siblings’ interactions are characterized by more competition, conflict, and even hostility, which in turn stimulates individuals’ short-term self-interest and makes them pay more attention to their own interests, which often leads to more risky behaviors (Menesini et al., 2010; Solmeyer et al., 2013), antisocial behavior (Criss and Shaw, 2005; Ensor et al., 2010; Buist and Vermande, 2014), and fewer prosocial behaviors (Kretschmer and Pike, 2010).

Sibling number shapes executives’ prosocial or antisocial preferences in their early life, which may greatly influence their social responsibility behavior during the adulthood period. The number of siblings affects the quality of sibling interaction by affecting the parents’ investment and the allocation of family resources, and this early experience of sibling interaction is internalized into the executives’ prosocial or antisocial preference. When there is a smaller number of siblings, less sibling rivalry makes it easier to form a harmonious sibling interaction relationship, such as showing more care, helping each other, and sharing with each other, which increases the executives’ prosocial tendency. However, when the number of siblings is relatively larger, the executives have to obtain family resources through competition in their childhood. In this case, the early family sibling interaction is mainly dominant by competition and conflict. Such conflictual sibling relationships aggravate the executives’ short-term self-interest and weaken their prosocial preferences.

Consistent with upper echelons theory, corporate activities are the reflection of top executives’ unique personalities and cognitive biases, and are significantly influenced by individual executives (Hambirck and Mason, 1984; Hambrick, 2007). Corporate social responsibility is a key business behavior, so firms’ CSR strategic decisions will be greatly influenced by the personal recognitive preferences of corporate executives (Plöckinger et al., 2016; Cronqvist and Yu, 2017). Therefore, executives’ prosocial biases, resulting from early harmonious experiences of sibling interaction with a smaller sibling number, prompts their prosocial behaviors and exerts positive effects on corporate CSR behavior. While executives’ antisocial preferences, stemming from conflictual sibling interaction experiences with a larger number of siblings, curb their prosocial behavior and reduce firms’ CSR behavior correspondingly.

Based on the above analysis, this paper proposes the following hypothesis:

**Hypothesis 1** There is an inverted U-shaped relationship between executives’ sibling number and corporate social responsibility behavior.

### The Moderating Effect of Sibling Differences

Sibling differences determine how children perceive the affection, warmth, competition, and conflict between siblings, and thus have an important impact on children’s cognitive abilities and behavioral tendencies in early childhood. In the face of fierce competition for family resources, children will try their best to show their own unique abilities and characteristics, so as to get special attention and treatment from parents and improve their ability to acquire family resources (Plomin and Daniels, 1987; Wang et al., 2009). Because children have individual differences, parents tend to adopt differential treatment according to their children’s individual characteristics (Tucker et al., 2003). This differential treatment negatively affects the quality of interaction between children and reduces the positive interaction between siblings (Stocker et al., 1989; Shanahan et al., 2008). Birth order and gender are natural differences that may influence the allocation of family resources and parental investment in different ways.

#### Birth Order

Generally, when parents are busy at work and do not have too much time and energy to take care of the younger children, the older children will naturally take the responsibility of caring for and teaching the younger siblings (Whiteman et al., 2011; Salmon et al., 2016). If there are fewer children in the family, there is less competition for family resources, and siblings are more likely to form harmonious interaction. In this sense, the eldest sibling will adopt more prosocial behaviors to the younger siblings, such as affection, help, sympathy, etc. On the contrary, a larger number of siblings leads to more fierce rivalry about family resources and parents’ investment. The elder siblings usually have a stronger ability of competition for resources (Freese et al., 1999), thus they are likely to get more household resources (Hotz and Pantano, 2015). In such a conflictual interaction typology, the older siblings maximize their own interests through their own age and power advantages, and their prosocial behaviors to the younger siblings will also be reduced correspondingly.

Therefore, when the number of executives’ siblings is relatively smaller, the early sibling interaction mainly involves a harmonious relationship. If an executive is the eldest, they will show stronger prosocial tendency toward the younger siblings. In the same way, they would also take prosocial behaviors to others, therefore, the company with a first-born executive might implement more CSR behavior than that with a later-born executive. Otherwise, when an executive has a relatively larger number of siblings in their childhood, the sibling interaction might be one with more competition and conflict. In this case, if an executive is the eldest, they would have a stronger ability to get more family resources and maximize their own interests. Thus, the first-born executives might exhibit fewer prosocial behaviors to their younger siblings, which further strengthens the negative influence that the sibling number has on the level of corporate social responsibility.

Therefore, we posit the following hypothesis:

**Hypothesis 2** The relationship between executives’ sibling number and corporate social responsibility will be strengthened in a company with a first-born executive compared to one with a later-born executive.

#### The Impact of Female Sibling

When the number of siblings is small, there is less competition for family resources and parents’ attention. Extant studies have suggested that females usually have much more prosocial tendencies and focus more on socialization (Buist, 2010; Cronqvist and Yu, 2017; Wu et al., 2019). When the executive is a male, the female siblings’ other-regarding bias may influence their male counterparts, increasing the prosocial behaviors to each other in the opposite sex siblings’ groups. However, when the number of siblings is larger, female siblings might be treated differently with fewer family resources and decreased attention from parents because of China’s “prefer boys over girls” traditional ideology. Female siblings are usually disadvantaged identities with poorer resource competitive ability. Thus, the degree of differential treatment of parents and unequal allocation of limited family resources is further aggravated (Plomin and Daniels, 1987; Wang et al., 2009). Therefore, the presence of female siblings increases sibling conflicts and rival for family resources and parents’ attention, strengthening the self-interest tendency between siblings and weakening their prosocial behaviors. If an executive is a woman and with female sibling(s), when there is a smaller number of siblings and less rivalry for family resources, siblings of the same sex are more likely to form in-group preferences and generate prosocial behaviors, such as sharing and helping (Buist, 2010). While a larger number of siblings induces intensified competition, since there is no gender disadvantage between executives and their female siblings, they have a stronger competitive ability to compete with each other and then strengthen the siblings’ conflict.

**FIGURE 1:**
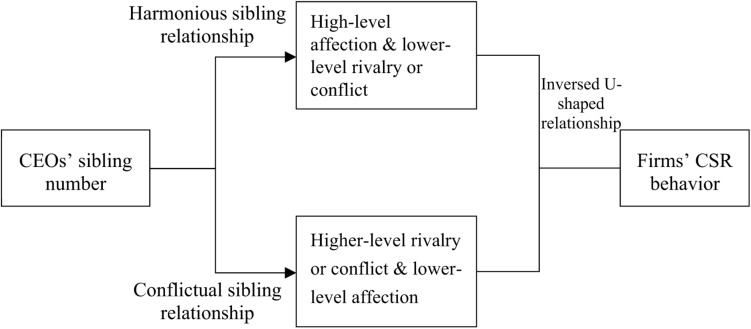
Relationship between CEOs’ sibling number and CSR behavior.

In the case of executives with a lower sibling number and family resources rivalry at an early age, female socialism of sister siblings may enhance executives’ prosocial behaviors. When executives have female siblings, the positive relationship between executives’ sibling number and corporate social responsibility performance would be stronger because of the positive impact that the prosocial preference of female siblings has on their male counterparts. However, under the circumstance that the number of executives’ siblings is larger and the allocation of family resources is unequal, the presence of female siblings might further increase their competition and conflict due to the unequal treatment. Hence, the presence of female siblings of executives strengthen the negative impact of the number of executives’ siblings on CSR behavior. The relationship between executives’ sibling number and CSR behavior are shown in Figure 1.

Therefore, we further propose the flowing hypothesis:

**Hypothesis 3** The relationship between executives’ sibling number and corporate social responsibility behavior will be strengthened when an executive has female sibling(s).

## Materials and Methods

### Data and Sample

In this paper, the Chinese A-share private listed companies on the Shanghai Stock Exchange and Shenzhen Stock Exchange from 2014 to 2017 were taken as data samples. Due to the fact that the corporate social responsibility of state-owned enterprises (SOEs) is largely subject to government administrative intervention, and the executives are also appointed by the government, the CSR behavior of SOEs is largely influenced by government political tasks, rather than executive personal experience. It is difficult to exactly investigate the relationship between executives’ personal traits and CSR performance. Therefore, we mainly chose Chinese private enterprises as the research samples. Our sample data starts from 2014, because in November 2013 the Third Plenary Session of the 18th Central Committee of the CPC in China puts forward new requirements for corporate social responsibility, which marks that CSR has reached a new stage for all companies.

We exclude ST and ST^∗^ samples, which refer to companies that have received special treatment because of two consecutive years of losses to avoid financial abnormality. We further eliminate financial listed companies because of their high level of leverage. And samples with missing data of executives’ siblings were also excluded. Finally, 1496 executives of sample companies have siblings. The data of corporate social responsibility (CSR) behavior is obtained from HeXun Website, which specializes in securities investment and advisory services, and discloses social responsibility ratings of all Chinese listed companies for many years. The data of executives’ siblings and other control variables is all from China Stock Market and Accounting Research Database (CSMAR) database excepting the data of company age from WIND database. In order to avoid the influence of extreme values, we winsorized all the continuous variables on the 1% level.

### Variable Definition

#### Corporate Social Responsibility Behavior

According to Long et al. (2020a), corporate social responsibility (CSR) behavior is mainly measured by the CSR ratings disclosed by the HeXun Website. The CSR ratings released by the HeXun Website include five aspects, namely, shareholders, employees, supply chain, the environment, and public benefit. The original data of CSR ratings is mainly from the corporate social responsibility report and annual report of Chinese listed companies. Therefore, such CSR ratings objectively and comprehensively measure corporate social responsibility behavior, even for companies who do not disclose in the CSR report. It ensured that our empirical research acquired a large sample size. Each sample company gets a CSR rating, and a higher CSR rating means more socially responsible corporate behavior.

#### Executives’ Sibling Number

There is not a database directly with information related to executives’ siblings, so we sorted out the available data through several steps. First, we obtained senior executives’ kinship data from CSMAR database, and excluded data other than siblings (e.g., parent, children) to get executives’ sibling data. Then we only chose executives’ personal biographical information and tenure information from the Senior Executives’ Personal Characteristics Database of CSMAR based on the position details. Further, the executives’ sibling data was gained by matching executives’ sibling data with executives’ personal biographical information. Finally, we calculated executives’ sibling number except for the executive himself/herself.

#### Executives’ Birth Order

We further eliminated the samples of executives with twin siblings. The missing data of siblings’ personal information were also dropped to obtain executives’ birth order data. According to executives’ and their siblings’ ages, we ranked their birth order. According to Campbell et al. (2019), we divided executives’ birth order into three categories, first-born, middle-born, and last-born. Specifically, the first-born group meant that the executives are the eldest in the family children group, while the last-born group refers to the executives who are the youngest. The middle-born group contains those executives with any other age.

#### The Impact of Female Sibling

In order to examine the moderating effect of female sibling(s), we divided the samples into two groups based on whether executives have female siblings or not. If an executive has at least one female sibling, then we mark it as 1. If the executive has no female sibling(s), this would be assigned as 0.

#### Control Variables

With reference to prior studies on CSR, we added some control variables to avoid the regression bias, including executive-, board-, and firm-level. The executive-level control variables mainly consist of the gender, age, degree, tenure, and shareholding ratio. Wu et al. (2019) suggests that female executives are more other-regarding, and pay more attention to philanthropy. In this study, we use dummy variables to control for the influence of executives’ gender. Additionally, previous studies also regard executives’ age, degree, tenure, and equity ownership as potential factors which may influence their CSR behaviors (Petrenko et al., 2016; Cronqvist and Yu, 2017).

The board-level control variables include board independence, the ratio of female directors, and directors with overseas background on the board, respectively. Board independence (Bi) helps to consider the interests of various external stakeholders, and improve executives’ CSR decision (Jain and Jamali, 2015). We use the proportion of independent directors to measure board independence. The ratio of female directors (Feratio) is measured by the proportion of female directors on board. The ratio of directors with overseas background is the proportion of directors who have overseas experience. Previous research has suggested the significant impact of female directors and directors with overseas backgrounds on CSR (e.g., Setó-Pamies, 2015; McGuinness et al., 2017; Hao et al., 2019).

The firm-level control variables constitute the firms’ profitability (ROA), firm size (Size), financial leverage (Lev), firm value (TQ), and firm age (FA). (1) Prior studies show that firm profitability may influence CSR practices (Sierra et al., 2013; Branco et al., 2014). When a company is profitable, more capital and resources are more likely to be invested in CSR issues. Firm profitability is measured as the ratio of net profit to the total assets. (2) Larger firms more easily receive wider attention, and they tend to focus more on CSR issues (Setó-Pamies, 2015). We use the natural logarithm of focal firm’s total assets to measure firm size. (3) Since extant studies have indicated CSR may be influenced by the leverage (Zorio et al., 2013; Xie et al., 2019), we also control for the possible effect of leverage. The asset-liability ratio (Lev) is measured as the total liabilities divided by total assets. (4) The firm value is calculated by the total market value divided by total assets (Rezende et al., 2019). Firms with higher values are more likely to obtain capital from investors in the capital market, and focus more on CSR to satisfy investors’ demands. (5) Referring to Lin et al. (2015), we use the number of years from its establishment to the sample year to measure firm age.

In addition, we control for the level of regional economic development in line with Wei et al. (2017), to avoid its influence on CSR. The fixed effect of industry and year are also controlled in the regression process. In particular, the industries were identified according to the Industry Classification Guidance of Listed Companies released by China Securities Regulatory Commission in 2012. Table 1 reports the detailed definition of all the variables.

**TABLE 1:** Variable definition.

**Variables**			**Definition**
Dependent variable		CSR	CSR ratings score disclosed by HeXun Website
Independent variable		Sibnum	Executives’ sibling number
Moderating variable		Birth order	Executives’ birth order (e.g., first-born, middle-born, and last born)
		Female sibling(s)	If an executive has female sibling(s), we mark it as 1, otherwise 0
Control variable	Executive-level	Age	Executives’ age
		Degree	Executives’ degree
		Tenure	The time spent in the executives’ position is measured in months
		Duality	Whether an executive is also the chairman and CEO, if “yes” marked 1, otherwise 0
		Shareratio	Number of shares held by the executives/total share capital
	Board-level	Bi	Number of independent directors/number of directors
		Feratio	Number of female directors/number of directors
		Overatio	Number of directors with overseas background/number of directors
	Firm-level	ROA	Net profit/total assets * 100
		Size	The natural log of total assets
		Lev	Total liabilities/total assets
		TQ	Market value/total assets
		FA	Firm age
	Region factors	ED	Per capita GDP of the region

### Model

According to the research hypothesis, we build model (1) to test the impact of executives’ sibling number on corporate social responsibility behaviors.

(1) $$C\,S\,R=\alpha +\beta_{1}*S\,i\,b\,n\,u\,m+\beta_{2}*S\,i\,b\,n\,u\,m^{\wedge }\,2+\gamma *C\,o\,n\,t\,r\,o\,l+\varepsilon$$

Where α is the constant, β_1_ and β_2_ are the estimated coefficients of the executives’ sibling number and its square terms, and γ is the estimated coefficient of the control variables. *Control* represents all control variables and ε is the residual. Year and Industry are dummy variables added into the model.

In order to test the moderating effect of executives’ birth order and gender composition, we mainly use the group method in the regression process. In line with the three groups of executives’ birth order, this paper re-estimates model (1) in the above three groups to test the impact of the birth order on the relationship between executives’ sibling number and CSR performance, separately. Similarly, this study uses the same regression method to re-estimate the model (1) in both the same-sex group and opposite-sex group, to examine the moderating effect of siblings’ gender composition.

## Results

### Descriptive Statistics and Correlation Analysis

Table 2 reports descriptive statistics of the main variables. The average CSR rating of the sample companies is 23.06, the standard deviation is 11.12, indicating that the performance of different companies in terms of social responsibility varies greatly among sample companies. The minimum value of the CSR rating is just −0.92, which suggests that the social responsibility of individual companies is still in a low level. The average number of executives’ siblings is 1.547, and the standard deviation is 0.963, showing that there is a small gap in the number of executives’ siblings in different companies.

**TABLE 2:** Descriptive statistics.

**Descriptive statistics of variables**							
**Variable**	**N**	**Mean**	**Median**	**SD**	**Range**	**Min**	**Max**
CSR	1,496	23.06	22.36	11.12	73.64	−0.92	72.72
Sibnum	1,496	1.55	1	0.96	7	1	8
Gender	1,496	0.93	1	0.26	1	0	1
Age	1,496	49.88	50	6.48	34	32	66
Degree	1,339	3.31	3	0.95	4	1	5
Duality	1,496	0.71	1	0.45	1	0	1
Tenure	1,496	52.69	44	35.17	149	1	150
Sharatio	1,475	18.77	14.96	17.46	60.87	0	60.87
Bi	1,496	0.41	0.4	0.08	0.35	0.27	0.63
Feratio	1,496	0.17	0.14	0.13	0.50	0	0.5
Overatio	1,496	0.11	0.1	0.12	0.55	0	0.55
ROA	1,496	0.06	0.05	0.05	0.29	−0.07	0.21
Size	1,496	21.62	21.56	0.94	4.47	19.94	24.41
Lev	1,496	0.34	0.31	0.18	0.77	0.046	0.82
TQ	1,496	3.61	2.94	2.42	12.01	0.54	12.55
FA	1,496	16.04	16	4.53	30	6	36
ED	1,496	7.54	7.44	2.33	10.29	2.61	12.90

From the descriptive statistics of the control variables, 92.8% of the executives in the sample companies are male, and the proportion of female executives is very small. The average age of the executives is 49.88 years old. The average degree is 3.31, and the median is 3, indicating that more than half of the executives have a bachelor degree or above. There are about 71.1% sample companies where the executives also hold the post of chairman in the board of directors. The percentage of independent directors’ ratio on the board is 0.405, showing that the proportion of independent directors in the sample company is slightly higher than the standard required by law, but basically remains at the compliance level. The percentage of female directors is 0.169, and the percentage of directors with overseas backgrounds is 0.109, which suggests that the proportion of female directors and directors with overseas background is still low and needs to be further improved. Descriptive statistics of other variables are shown in Table 2. Table 3 reports the correlation coefficients of the main variables. The results show that the correlation coefficients between the main variables are relatively small, and there is no multicollinearity problem.

**TABLE 3:** Correlations.

	**CSR**	**Sibnum**	**Gender**	**Age**	**Degree**	**Duality**	**Tenure**	**Sharatio**	**Bi**	**Feratio**	**Overatio**	**ROA**	**Size**	**Lev**	**TQ**	**FA**	**ED**
CSR	1																
Sibnum	−0.046*	1															
Gender	0.0385	0.0726***	–0.113														
Age	0.0218	0.0945***	–0.0113	1													
Degree	0.088***	−0.0676**	0.0767***	−0.0895***	1												
Duality	−0.0466*	0.0478*	0.1357***	0.341***	0.0049	1											
Tenure	–0.0209	–0.0133	0.0506*	0.0751***	0.0208	0.0178	1										
Sharatio	−0.0573**	−0.0567**	–0.0092	0.1104***	–0.0322	0.406***	−0.0607**	1									
Bi	–0.0001	0.0207	−0.0901***	–0.0088	–0.0003	–0.0191	0.0004	0.0226	1								
Feratio	0.01	0.0078	−0.2054***	0.0649**	–0.0139	0.033	–0.0112	0.0907***	0.0435*	1							
Overatio	0.0884***	–0.0205	0.0465*	–0.0394	0.106***	–0.0175	0.0659**	–0.0395	0.0594**	–0.0086	1						
ROA	0.461***	−0.0791***	0.0240	0.0437*	–0.0133	–0.0017	−0.175***	0.0848***	0.0157	–0.0060	0.0434*	1					
Size	0.149***	0.0794***	0.0410	–0.0005	0.0452*	−0.0682***	0.289***	−0.244***	−0.0462*	−0.0680***	0.1866***	−0.0805***	1				
Lev	−0.119***	0.1014***	0.0512**	–0.0379	0.009	0.0322	0.13***	−0.117***	–0.0279	−0.0501*	0.0356	−0.337***	0.528***	1			
TQ	0.0928***	−0.087***	–0.0328	0.0062	0.0118	0.0461*	−0.198***	0.169***	0.0339	0.0176	–0.0097	0.346***	−0.523***	−0.45***	1		
FA	0.0053	0.0247	0.0664**	0.146***	0.0176	−0.0525**	0.208***	–0.0192	–0.0278	0.0261	0.0071	–0.0418	0.147***	0.0803***	−0.134**	1	
ED	0.0223	−0.0953***	0.0156	–0.0312	0.0696**	0.0537**	–0.0070	0.0828***	0.0788***	–0.0180	–0.0268	–0.0091	0.0134	–0.0240	0.0932***	0.0262	1

***, **, and * are significant at the 1, 5, and 10% levels, respectively.

### Regression Results Analysis

#### Main-Effect Analysis

Table 4 reports the multiple regression results of the number of executives’ siblings and the level of corporate social responsibility. According to the model (1) constructed above, the first column in Table 4 shows the regression results of the number of executives’ siblings and the level of CSR under the control of the executives’ personal characteristic variables and the dummy variables of the industry and the year. On this basis, the second column adds control variables at the level of board characteristics. Finally, the third column further controls the variables at firm-level, including profitability (ROA), firm size (Size), asset-liability ratio (Lev), firm value (TQ), and firm age (FA). It can be seen from the adjusted R square of the three regression results that the model’s fitting degree is also improved after the control variables are gradually increased.

**TABLE 4:** Regression results of CEOs’ sibling number and CSR performance.

	**(1)**	**(2)**	**(3)**
**VARIABLES**	**CSR**	**CSR**	**CSR**
Sibnum	1.2559	1.5266	1.8607**
	(1.20)	(1.47)	(2.02)
Sibnum^2	−0.3335**	−0.3807**	−0.3816***
	(−1.98)	(−2.28)	(−2.60)
Gender	2.8364***	3.0359***	1.7710**
	(2.64)	(2.64)	(2.00)
Age	0.0945*	0.0972**	0.0209
	(1.91)	(2.00)	(0.54)
Degree	0.9594***	0.8606***	0.8246***
	(3.10)	(2.74)	(3.10)
Duality	−1.0523	−1.0517	0.1584
	(−1.26)	(−1.26)	(0.22)
Tenure	−0.0141	−0.0151	−0.0030
	(−1.47)	(−1.58)	(−0.32)
Sharatio	−0.0201	−0.0202	−0.0395**
	(−1.19)	(−1.20)	(−2.55)
Bi		−2.5079	−2.3280
		(−0.67)	(−0.70)
Feratio		4.2757*	4.7171**
		(1.66)	(2.03)
Overatio		7.7040***	2.5325
		(2.88)	(1.08)
ROA			1.2354***
			(17.10)
Size			1.8253***
			(3.72)
Lev			−5.4541***
			(−2.96)
TQ			−0.4376***
			(−3.18)
FA			0.0314
			(0.58)
ED			−0.0549
			(−0.42)
Constant	11.8717**	11.5090**	1321
	(2.57)	(2.23)	0.3028
Observations	1321	1321	16.99
Adjusted R-squared	0.0472	0.0543	0.3045
F	3.884	3.653	16.44

***, **, and * are significant at the 1, 5, and 10% levels, respectively, the t-value is in parentheses.

The results of Column1 and Column 2 show that the estimated coefficients between the square item of the number of executives’ siblings and the CSR behavior are −0.3335 and −0.3807, respectively, which is significant at the confidence level of 5%. Therefore, the inversed U-shaped relationship between the number of executives’ siblings and the CSR behavior is preliminarily confirmed. The result of Column 3 shows that the estimated coefficient of executives’ sibling number and firms’ CSR behavior is 1.8607, which is significant at the level of 5%, while the estimated coefficient of the square of executives’ sibling number and CSR behavior is −0.3816 and significant under the confidence level of 1%. Therefore, the research hypothesis 1 is supported.

According to Haansr et al. (2016), the balance point of the inversed U-shaped relationship between executives’ sibling number and CSR behavior is about 2.44 (e.g., the balance point is calculated as the estimated coefficient of executives’ sibling number divided by twice the square term coefficient in absolute value). That is, as the number of executives’ siblings is less than 2.44, executives’ sibling numbers will positively promote corporate social responsibility performance; while if the number of executives’ siblings exceeded 2.44, the CSR performance decreased with the increase of the executives’ sibling number. The results suggest that when the executives’ sibling number is within a certain range, the early interaction between siblings was more likely to give priority to a harmonious relationship. Thus, the increase in the number of siblings gradually enhanced executives’ prosocial tendencies with an early harmonious family relationship, which may significantly promote executives’ CSR behavior. However, when the executive’s sibling number is over this scope, the rivalry and conflict in early sibling interactions was increased. Under this circumstance, siblings form a strong personal self-interest to maximize their own family resources, and the prosocial behavior preference decreased correspondingly, thereby reducing the CSR behavior in the executives during adulthood.

#### Moderating Effect Analysis

Table 5 reports the regression results after the grouping according to birth order. Column 1 shows the regression result of executives’ sibling number and firms’ CSR performance when an executive is the first-born. The estimated coefficient of executives’ sibling number and firms’ CSR performance is 4.0855, and the coefficient of the square term is −0.6580, both significant at 5% level. Compared with the main effect, the balance point (3.10) of the inversed U-shaped relationship between executives’ sibling number and CSR performance is much higher when an executive is the eldest. This result suggests that the negative effect of executives’ sibling number on firms’ CSR performance will be delay when an executive is first-born. Column 2 and Column 3 separately show the regression results in the case that an executive is middle-born or last-born. Through the comparison, we find that executives’ birth orders had no significant influence on the relationship between the number of executives’ siblings and firms’ CSR performance when executives were middle-born and last-born.

**TABLE 5:** Moderating effect of CEO birth order.

	**(1)**	**(2)**	**(3)**
	** First-born**	**Middle-born**	**Last-born**
Sibnum	4.0855**	3.8676	2.4265
	(2.42)	(0.81)	(0.88)
Sibnum^2	−0.6580**	−0.4426	−0.6493
	(−2.48)	(−0.57)	(−1.28)
Gender	3.1229	−4.7650	1.8781
	(1.41)	(−1.30)	(0.57)
Age	−0.0249	−0.1103	0.0760
	(−0.25)	(−0.60)	(0.51)
Degree	1.1370*	0.6908	0.6296
	(1.91)	(0.82)	(0.73)
Duality	2.7068	2.5314	0.8528
	(1.74)	(1.01)	(0.42)
Tenure	0.0002	0.0774***	−0.0226
	(0.01)	(3.02)	(−1.00)
Sharatio	−0.0224	0.0481	−0.0232
	(−0.64)	(0.76)	(−0.40)
ROA	1.1043***	1.2657***	1.2601***
	(7.14)	(7.52)	(6.80)
Size	2.1796**	−1.3402	2.3899*
	(2.43)	(−0.84)	(1.92)
Lev	−10.0338**	3.2707	−1.8878
	(−2.56)	(0.27)	(−0.30)
TQ	−0.1114	−0.4016	−0.9498*
	(−0.34)	(−0.60)	(−1.91)
Bi	0.0805	−8.1554	6.3782
	(0.01)	(−1.03)	(0.66)
Feratio	15.2193***	−4.6802	−7.4860
	(3.42)	(−0.58)	(−1.19)
Overatio	−6.2735	−6.0746	4.3905
	(−1.34)	(−0.88)	(0.63)
FA	0.2041	0.3501	0.1586
	(1.59)	(1.49)	(0.72)
ED	−0.3697	−0.0413	−0.6811*
	(−1.39)	(−0.10)	(−1.85)
Industry	YES	YES	YES
Year	YES	YES	YES
Constant	−37.3191	37.8494	−40.7554
	(−1.65)	(1.17)	(−1.39)
N	354	37	285
R^2^	0.2961	0.8470	0.2799
F	5.641	10.06	4.807

***, **, and * are significant at the 1, 5, and 10% levels, respectively, the t-value is in parentheses.

The results indicate that under the circumstance of lower sibling number and competition in early family resources, an executive has a stronger sense of responsibility and prosocial tendency when he or she is the eldest, which has a significant promoting effect on the CSR performance of the enterprise the executive is engaged in. However, when the executives’ sibling number is larger, the competition and conflict of family resources are fiercer in the early family life, thereby increasing the executives’ self-interest orientation. The first-born executives usually had a stronger power and ability advantages, which might further aggravate the sibling conflict and promote executives’ short-term self-interest tendency. Therefore, the first-born executives further reduce the firms’ CSR performance in the case of a larger executive’s sibling number. Hypothesis 2 is verified finally.

**FIGURE 2:**
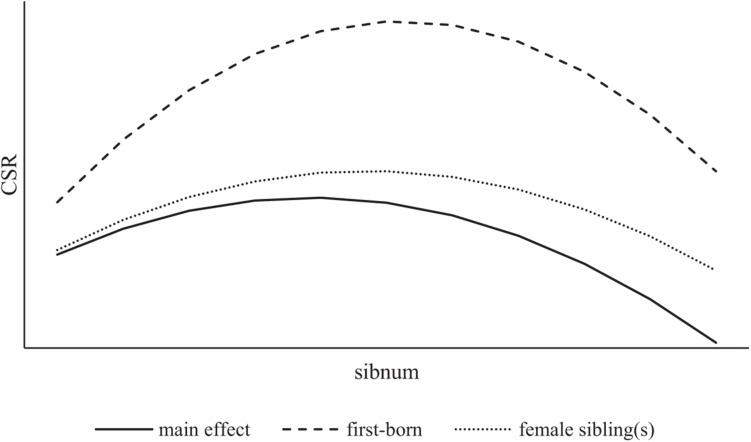
Moderating effect of birth order and female sibling(s).

Table 6 reports the moderating effect of female sibling(s). The results in column 2 show that the estimated coefficient of the number of executives’ siblings and its square is 2.0130 and −0.3546, respectively, and both significant at the 5% level, suggesting that the inversed U-shaped relationship between executives’ sibling number and firms’ CSR performance is strengthened when executives have female sibling(s). The estimated equilibrium point is 2.61, slightly higher than that of the main effect, indicating that the presence of executives’ female siblings delays the negative impact of the number of siblings on the CSR behavior to some extent. However, the inversed U-shaped relationship between executives’ sibling number and CSR is not supported when they are with female sibling(s). Therefore, Hypothesis 2 is supported. Figure 2 shows the relationship between executives’ sibling number and CSR and the moderating effect of executives’ birth order and female sibling(s).

**TABLE 6:** The moderating effect of female sibling(s).

	**(1)**	**(2)**
	**With female sibling(s)**	**Without female sibling**
Sibnum	2.0130*	1.7778
	(1.85)	(0.65)
Sibnum^2	−0.3546**	−0.0901
	(−1.97)	(−0.14)
Gender	3.0714**	1.5972
	(1.97)	(0.97)
Age	0.0719	−0.1151
	(1.23)	(−1.57)
Degree	0.1838	0.9972**
	(0.47)	(2.36)
Duality	−1.7578*	1.7889*
	(−1.77)	(1.73)
Tenure	−0.0157	0.0217*
	(−1.38)	(1.81)
Sharatio	−0.0667***	−0.0361
	(−2.89)	(−1.38)
ROA	1.1398***	1.3389***
	(12.39)	(13.07)
Size	0.6215	3.5134***
	(1.13)	(5.35)
Lev	−3.8094	−7.5452**
	(−1.44)	(−2.54)
TQ	−0.4650**	−0.2169
	(−2.07)	(−0.96)
Bi	1.1882	−5.0656
	(0.27)	(−1.01)
Feratio	3.6878	5.8626*
	(1.32)	(1.77)
Overatio	6.2668**	−7.2860**
	(2.15)	(−2.07)
FA	0.0699	−0.0936
	(0.85)	(−0.96)
ED	1.6123	−0.4341
	(1.37)	(−0.32)
Industry	YES	YES
Year	YES	YES
Province	YES	YES
Constant	−38.6506**	−50.4949**
	(−2.03)	(−2.42)
N	588	733
R^2^	0.4120	0.3409
F	7.856	7.528

***, **, and * are significant at the 1, 5, and 10% levels, respectively, the t-value is in parentheses.

### Robust Test

#### Sample Selection Bias

In order to address the possible bias of sample selection, we further use a propensity score matching (PSM) method to avoid the endogeneity. First, this study starts with a much boarder samples (executives with and without siblings), and there are 2013 initial samples. Then, we match each observation to one for which an executive has siblings based on executives’ gender, age, degree, duality (dummy variable, yes for 1, no for 0), ROA, Lev, TQ, and industry. The balance test results reported in Table 7 show that the standard bias of each variable between the treatment group and the control group is far less than 10%, suggesting that the matching results are acceptable (Smith and Todd, 2005). Through PSM, we only lost 27 from the total 2013 samples, which has relatively little influence on the subsequent regression results. Thus, we re-estimate the baseline model with the matched data, and the regression results are shown in Column 1 of Table 8. The results are similar with our baseline model even after PSM, and the inversed U-shaped relationship between executives’ sibling number and CSR is still supported when considering the endogeneity problem.

**TABLE 7:** PSM results.

**PSM estimates**			**Balance test**				
**Variables**	**Coefficient**	**z**	**Treated**	**Control**	**Bias (%)**	**t**	**p > | t |**
Gender	0.3999	2.35	0.9283	0.9357	–2.6	–0.77	0.443
Age	−0.0132	–1.77	49.796	49.871	–1.1	–0.27	0.786
Degree	−0.2553	–6.08	3.3281	3.293	2.8	0.86	0.388
Duality	0.3301	2.97	0.7100	0.6958	3.1	0.80	0.422
ROA	0.0288	2.43	5.7063	5.5246	4.1	1.05	0.294
Lev	−0.2969	–0.92	0.3312	0.3274	2.2	0.58	0.565
TQ	−0.0767	–3.56	3.6773	3.7324	–2.1	–0.59	0.555
Industry	−0.0397	–2.05	3.8692	3.8117	2.3	0.64	0.520
Constant	2.0512	4.42					
N	2013						
Pseudo R^2^	0.0303						

**TABLE 8:** Robust test result.

	**(1)**	**(2)**	**(3)**
	**CSR**	**CSR**	**CSR**
Sibnum	1.5474***	1.9092**	1.9648*
	(2.76)	(2.00)	(1.77)
Sibnum^2	−0.3276***	−0.3721**	−0.4220**
	(−2.73)	(−2.15)	(−2.09)
Gender	0.9318	1.1881	1.9901*
	(1.07)	(1.09)	(1.65)
Age	0.0468	0.0111	0.0241
	(1.29)	(0.24)	(0.47)
Degree	0.7209***	0.6272**	1.0666***
	(3.44)	(2.19)	(3.33)
Duality	0.9875*	0.1487	0.7873
	(1.71)	(0.21)	(1.01)
Tenure	0.0033	−0.0032	−0.0080
	(0.47)	(−0.39)	(−0.87)
Sharatio	−0.0427**	−0.0456***	−0.0431**
	(−2.54)	(−2.62)	(−2.25)
ROA	1.2676***	1.2490***	1.2263***
	(20.32)	(17.88)	(15.77)
Size	2.2799***	1.8080***	2.0503***
	(6.21)	(4.25)	(4.29)
Lev	−5.3835***	−5.6233***	−5.7030**
	(−3.09)	(−2.80)	(−2.55)
TQ	−0.4726***	−0.3777**	−0.4068**
	(−3.40)	(−2.34)	(−2.28)
Bi	−2.3607	−2.3973	0.6227
	(−0.77)	(−0.71)	(0.16)
Feratio	2.3761	4.1105*	6.0952**
	(1.28)	(1.93)	(2.58)
Overatio	1.7437	2.0511	−0.8575
	(0.87)	(0.91)	− 0.33)
FA	0.0478	0.0344	0.0529
	(0.94)	(0.54)	(0.73)
ED	−0.0022	0.3385	−0.1884
	(−0.02)	(0.37)	(−1.37)
Industry	YES	TES	YES
Year	YES	YES	YES
Region	−	YES	−
Constant	−43.3761***	−33.1075**	−38.8060***
	(−5.16)	(−2.37)	(−3.50)
Observations	1957	1,321	1054
(Adjust) R-squared	0.2831	0.3344	0.3007
F	23.07	11.87	15.15

***, **, and * are significant at the 1, 5, and 10% levels, respectively, the t-value is in parentheses.

#### Method Substitution

In order to test the robustness of our results, we further use panel data regression model with multidimensional fixed-effect to examine the impact of executives’ sibling number on CSR behaviors according to Xu et al. (2016). When considering region, industry, and year fixed effect simultaneously, Column 2 of Table 8 reports the regression results, which shows that the estimated coefficient of executives’ sibling number is 1.9092, and the coefficient of the square term is −0.3721, both significant under the confidence level of 5%. The regression results are basically the same with the main effect, suggesting that our research results have relatively strong robustness.

#### Random Sample Selection

With reference to Li et al. (2009), we randomly deleted 20% samples for the regression process to avoid sample selection bias. After repeated tests, it was found that the regression results and significance level have no significant changes. The third column in Table 8 reports one of the multiple regression results among them, indicating that the inverted U-shaped relationship between the number of CEOs’ siblings and the CSR performance remains stable.

### Further Study

#### Effect of Executives’ Famine Experience

An early life experience imprints persistently in individual’s subsequent perception of external conditions and events (Cronqvist et al., 2012; Bernile et al., 2017). Xu and Li (2016) proposed that executives’ early famine experience might increase their prosocial tendencies and enhance firms’ donation behavior. Thus executives with famine experience more easily show sympathy and affection to their siblings and even others. Similarly, these executives are prone to take more prosocial behaviors and improve the firms’ CSR performance where they work, no matter how many siblings. In other words, the relationship between executives’ sibling number and CSR behaviors might be weakened when they have early famine experience.

The Great Chinese Famine, during the period from 1959 to 1961, was a widespread famine which brought about great influences on economic, social, environmental, and other aspects. Such early famine experience deeply imprints on individuals’ minds and influences one’s cognition and decision-making in later life. Prior studies have provided evidence that the famine experience may influence an individual’s decisions on donation, risk, investment, etc. (Schoar and Zuo, 2017; Feng and Johansson, 2018). Therefore, we believe that executives’ famine experience may also impact their sibling interaction quality in their early family life and their prosocial bias. Then we further take the Great Chinese Famine, an exogenous event, to examine the moderating effect on the relationship between executives’ sibling number and CSR. According to Long et al. (2020b), we match executives birth year with the Great Chinese Famine during 1959–1961. If the period of famine happened during the childhood of the executives (0–14 years old), then these executives were considered to have famine experience, and we mark it as 1. Otherwise is 0.

Afterward, we re-estimated the model (1) both in groups of executives with and without famine experience. Table 9 reports the corresponding group regression results. The results suggest that the inversed U-shaped relationship between executives’ sibling number and firms’ CSR performance is strengthened when executives without famine experience but is not significant for executives with famine experience. The above results show the reverse, that executives’ famine experience may weaken the relationship between sibling number and CSR behavior. The reason why the results in Column 1 are not significant may be due to the fact that the proportion of executives with famine experience is small in the sample and the sibling number of these executives is also relatively concentrated.

**TABLE 9:** The influence of executives’ famine experience.

	**(1)**	**(2)**
	**Famine = 1**	**Famine = 0**
Sibnum	0.8997	2.2154**
	(0.51)	(2.02)
Sibnum^2	−0.2598	−0.4222**
	(−0.80)	(−2.18)
Gender	0.2776	1.6928
	(0.17)	(1.25)
Age	0.1852	0.0885
	(1.26)	(1.30)
Degree	1.0227**	0.8218**
	(2.24)	(2.33)
Duality	0.9892	−0.2025
	(0.68)	(−0.25)
Tenure	−0.0305**	−0.0008
	(−2.18)	(−0.08)
Sharatio	−0.0204	−0.0487**
	(−0.66)	− 2.40)
ROA	1.5387***	1.1648***
	(12.13)	(14.15)
Size	1.8623***	1.8760***
	(2.72)	(3.59)
Lev	−3.2981	−5.7762**
	−0.97)	(−2.43)
TQ	−0.3586	−0.4795**
	(−1.37)	(−2.43)
Bi	2.8719	−3.5857
	(0.48)	(−0.89)
Feratio	3.7357	4.3590*
	(0.95)	(1.69)
Overatio	3.8121	1.8467
	(0.94)	(0.69)
FA	0.1562	−0.0215
	(1.50)	(−0.28)
ED	−0.4620**	0.0624
	(−2.22)	(0.42)
Industry	YES	YES
Year	YES	YES
Constant	−44.2260**	−34.6321***
	−2.36)	(−2.86)
Observations	265	1056
Adjusted R-squared	0.4781	0.2801
F	9.062	13.82

***, **, and * are significant at the 1, 5, and 10% levels, respectively, the t-value is in parentheses.

#### Effect of Confucian Culture

Confucianism is the traditional culture of China, and Confucian social ethics stresses the importance of family, which requires individuals to respect to the parents, and show love and affection to siblings. In the regions with strong Confucian culture, individuals are more likely to be influenced by the family ethics culture and focuses much more on family responsibility. Under this circumstance, individuals may experience harmonious sibling relationships and fewer sibling conflicts in their early family life, regardless of the number of siblings. In this sense, Confucian culture weakens the relationship between an individual’s sibling number and prosocial behavior.

With reference to Gu (2015), we first collected data of the number of Confucian schools in each region from the local Chronicles of the Qing Dynasty, and then calculated the average value of all the samples. After that, we divided the samples into two groups, and regard the regions higher than the mean as strong Confucian culture groups, while the others were regarded as weak Confucian culture groups. We re-estimated the model (1) in the two groups to examine the effect of Confucian culture, and the regression results are shown in Table 10. The results in Column 1 suggest that the relationship between executives’ sibling number and CSR behavior is weaker in the regions with strong Confucian culture.

**TABLE 10:** The influence of Chinese Confucian culture.

	**(1)**	**(2)**
**VARIABLES**	**Culture = 1**	**Culture = 0**
Sibnum	1.8014	1.6930
	(1.33)	(1.26)
Sibnum^2	−0.4282**	−0.2386
	(−2.01)	(−0.89)
Gender	0.8465	2.6581***
	(0.37)	(3.01)
Age	0.0634	−0.0037
	(0.95)	(−0.07)
Degree	0.4251	0.9165***
	(0.96)	(3.02)
Duality	1.5703	−0.8147
	(1.38)	(−0.90)
Tenure	0.0076	−0.0112
	(0.53)	(−0.90)
Sharatio	−0.0344	−0.0582***
	(−1.32)	(−3.46)
ROA	1.2712***	1.2459***
	(9.51)	(14.39)
Size	3.2498***	0.6324
	(3.92)	(1.15)
Lev	−9.0933***	−2.8368
	(−3.25)	(−1.20)
TQ	−0.2408	−0.7191***
	(−1.01)	(−4.40)
Bi	−5.4977	1.5114
	(−0.92)	(0.41)
Feratio	−0.8432	8.4635***
	(−0.26)	(2.66)
FA	−0.0336	0.0992
	(−0.36)	(1.57)
ED	−0.0454	−0.0120
	(−0.15)	(−0.10)
Constant	−60.7102***	−9.5742
	(−3.15)	−0.73)
Observations	577	744
Adjusted R-squared	0.2711	0.3653
F	8.653	

***, **, and * are significant at the 1, 5, and 10% levels, respectively, the t-value is in parentheses.

## Conclusion and Discussion

### Conclusion

This paper empirically tested the influence of the number of executives’ siblings on the corporate social responsibility performance by taking Chinese A-share private listed companies from 2014 to 2017 as data samples. The empirical results show that there is an inversed U-shaped relationship between executives’ sibling number and firms’ CSR performance. In other words, when the number of executives’ siblings is small, there is less rivalry for early family resources and parents’ investment, resulting in a harmonious early sibling interaction relationship. This early warm family experience increased executives’ prosocial tendency, thereby improving the executives’ CSR behavior during their adulthood. However, when the executives’ sibling number exceeds a certain range, the increase in the number of siblings would decrease executives’ CSR behavior. Further research shows that when an executive is the oldest among siblings or with female sibling(s), the influence of the number of executives’ siblings on firms’ CSR behavior will be strengthened.

### Implication and Limitations

This paper has three theoretical implications for the existing research: First, the research on family sibling effect is extended from the field of social psychology to the business practice. This paper enriches the studies on the influence of executives’ early life experience on corporate strategic decision-making through prosocial cognitive formation. The research on family sibling effect in the field of social psychology mainly focuses on the influence of sibling effect on individual’s internal psychology or external behavior. As an individual, an executive’s early family life would inevitably affect his/her cognitive formation and behavior preference, which will be brought forward to the strategic decision of the enterprises they are involved in. Specifically, the number of executives’ siblings, the quality of siblings’ interactions, and the birth order all have an impact on their sense of responsibility and prosocial behavior, and then affect their decisions on firms’ CSR issues.

Second, it expands the research on the driving factors of CSR and finds a new driving factor of CSR behavior. Existing research has explored the driving factors of CSR from the perspective of executives’ cognitive traits (McCarthy et al., 2017; Tang et al., 2018; Hegde and Mishra, 2019), but few studies focused on the influence of executives’ early family life experience on firms’ CSR behavior. From the perspective of executives’ early cognitive traits, this paper investigates the influence of the executives’ sibling effect on the social responsibility behavior of the company they served in the adulthood. Our research shows that the number of executives’ siblings in the early family domain shapes the personal prosocial tendency by influencing their sibling relationship, thereby determining the firms’ CSR behavior.

Thirdly, this paper enriches the research on the influence of executives’ personal experience and early life on enterprises’ performance. Many studies have focused on the influence of executives’ demographic characteristics, work experience, and values on CSR performance (McGuire et al., 2003; Deckop et al., 2006; Tang et al., 2015, 2018), but neglected the important role of the early family environment on executives’ cognitive preference and corporate decision-making. From the perspective of executives’ early family interaction, this paper studied how the siblings affect corporate social responsibility by influencing executives’ prosocial tendencies. The study extends the influencing factors of corporate social responsibility behavior from the perspective of the executives’ early family domain, which is conducive to a profound understanding of the influence of the executive’s early cognitive formation on their business behavior and decision-making.

The study also has the following limitations: (1) the number of siblings is just one of the key factors of affecting the interaction quality between siblings, and other factors, such as family economic level and parents’ attitudes toward the children will also have an impact on childhood sibling relationship. Therefore, it is necessary to deeply explore other family factors influencing individuals’ early cognitive formation. (2) There are many traditional cultures that will affect individuals’ cognition and behavioral tendencies. Further research could investigate the influence of various Chinese cultural context factors. (3) This paper only studied the influence of executives’ sibling traits on CSR behavior, so future research can further consider the influence of executives’ early cognitive preferences on other business behaviors.

## Data Availability Statement

The datasets presented in this article are not readily available because they contain entrepreneurs’ personal information. Requests to access the datasets should be directed to the corresponding authors.

## Ethics Statement

The studies involving human participants were reviewed and approved by the Ethics Committee of Nankai University. The patients/participants provided their written informed consent to participate in this study.

## Author Contributions

MZ provided the overall conceptual model and wrote the original manuscript. GR modified the manuscript and provided ideas and suggestions for revision. LZ provides supplementary data and analysis for the revision. All authors contributed to the article and approved the submitted version.

## Conflict of Interest

The authors declare that the research was conducted in the absence of any commercial or financial relationships that could be construed as a potential conflict of interest.
